# Geranylgeranyl diphosphate synthase inhibitor and proteasome inhibitor combination therapy in multiple myeloma

**DOI:** 10.1186/s40164-022-00261-6

**Published:** 2022-02-09

**Authors:** Staci L. Haney, Michelle L. Varney, Jacob T. Williams, Lynette M. Smith, Geoffrey Talmon, Sarah A. Holstein

**Affiliations:** 1 Division of Oncology and Hematology, Department of Internal Medicine, University of Nebraska Medical Center, Omaha, NE 68198 USA; 2 Department of Biostatistics, University of Nebraska Medical Center, Omaha, NE USA; 3 Department of Pathology and Microbiology, University of Nebraska Medical Center, Omaha, NE USA

## Abstract

**Background:**

Multiple myeloma (MM) remains an incurable malignancy, despite the advent of therapies such as proteosome inhibitors (PIs) that disrupt protein homeostasis and induce ER stress. We have pursued inhibition of geranylgeranyl diphosphate synthase (GGDPS) as a novel mechanism by which to target protein homeostasis in MM cells. GGDPS inhibitors (GGSI) disrupt Rab geranylgeranylation, which in turn results in perturbation of Rab-mediated protein trafficking, leading to accumulation of intracellular monoclonal protein, induction of ER stress and apoptosis. Our lead GGSI, RAM2061, has demonstrated favorable pharmacokinetic properties and in vivo efficacy. Here we sought to evaluate if combination therapy with GGSI and PI would result in enhanced disruption of the unfolded protein response (UPR) and increase anti-MM efficacy.

**Methods:**

MTT assays were conducted to evaluate the cytotoxic effects of combining RAM2061 with bortezomib in human MM cells. The effects of RAM2061 and/or PI (bortezomib or carfilzomib) on markers of UPR and apoptosis were evaluated by a combination of immunoblot (ATF4, IRE1, p-eIF2a, cleaved caspases and PARP), RT-PCR (ATF4, ATF6, CHOP, PERK, IRE1) and flow cytometry (Annexin-V). Induction of immunogenic cell death (ICD) was assessed by immunoblot (HMGB1 release) and flow cytometry (calreticulin translocation). Cell assays were performed using both concurrent and sequential incubation with PIs. To evaluate the in vivo activity of GGSI/PI, a flank xenograft using MM.1S cells was performed.

**Results:**

Isobologram analysis of cytotoxicity data revealed that sequential treatment of bortezomib with RAM2061 has a synergistic effect in MM cells, while concurrent treatment was primarily additive or mildly antagonistic. The effect of PIs on augmenting RAM2061-induced upregulation of UPR and apoptotic markers was dependent on timing of the PI exposure. Combination treatment with RAM2061 and bortezomib enhanced activation of ICD pathway markers. Lastly, combination treatment slowed MM tumor growth and lengthened survival in a MM xenograft model without evidence of off-target toxicity.

**Conclusion:**

We demonstrate that GGSI/PI treatment can potentiate activation of the UPR and apoptotic pathway, as well as induce upregulation of markers associated with the ICD pathway. Collectively, these findings lay the groundwork for future clinical studies evaluating combination GGSI and PI therapy in patients with MM.

**Supplementary Information:**

The online version contains supplementary material available at 10.1186/s40164-022-00261-6.

## Background

Multiple myeloma (MM) is a blood cancer caused by the malignant proliferation of clonal plasma cells in the bone marrow. MM accounts for 10% of all hematological cancers and approximately 35,000 new cases will be diagnosed in 2021. MM cells produce and secrete large quantities of monoclonal proteins (MP), which can be detected in the peripheral blood. To alleviate endoplasmic reticulum (ER) stress from the continuous manufacturing of MP, MM cells express near maximal levels of unfolded protein response (UPR) associated proteins and therefore are particularly sensitive to activation of the pro-apoptotic arm of the UPR [1, 2]. Disruption of the machinery regulating protein homeostasis and the resulting induction of ER stress-associated apoptosis serves as a targeted therapeutic strategy against MM [3–5]. Current front-line and relapsed/refractory treatments for MM include proteasome inhibitors (PIs), which work by blocking the proteasome from recycling misfolded proteins, leading to ER stress and apoptosis [6–8]. The FDA approved the first-in-class PI, bortezomib, in 2003 followed by the second-generation PI, carfilzomib, in 2012. While PIs have proven very beneficial for the treatment of MM, development of relapsed/refractory MM remains a significant clinical challenge [9–11] and necessitates the need for novel therapies.

As an alternative strategy by which to target protein homeostasis in MM, we have focused on the development of geranylgeranyl diphosphate synthase (GGDPS) inhibitors (GGSIs) [12–21]. In the isoprenoid biosynthetic pathway (IBP), GGDPS catalyzes the synthesis of the isoprenoid donor (GGPP) which in turn is covalently linked to the target Rab proteins by the enzyme geranylgeranyl transferase (GGTase) II. Rabs regulate nearly all intracellular trafficking events and require geranylgeranylation in order to be properly localized and functional [22]. We have shown that agents that impair Rab geranylgeranylation induce MM cell death by disrupting the trafficking of MP, resulting in induction of the UPR and apoptosis [23–25]. We have previously reported preclinical studies with our lead GGSI, RAM2061, which demonstrated the agent’s metabolic stability, prolonged half-life, systemic distribution, in vivo disruption of geranylgeranylation and anti-tumor efficacy in a mouse MM xenograft model [24].

We hypothesized that the combination of GGSI and PI therapy would result in enhanced activation of the UPR and increase anti-MM efficacy. Here we demonstrate that combination treatment with RAM2061 and PI (bortezomib or carfilzomib) enhances UPR activation and MM cell death in vitro when compared to single drug treatment, and that these effects are dependent on the timing of drug exposure. In addition, combination therapy induces activation of the immunogenic cell death pathway (ICD). Furthermore, combination GGSI and bortezomib treatment significantly slowed in vivo tumor growth and lengthened survival relative to single drug treatments in a mouse MM xenograft model. Collectively, these studies are the first to demonstrate the feasibility and therapeutic benefit of combining GGSI and PIs and lay the foundation for future clinical studies.

## Methods

### Chemicals

Dr. David Wiemer at the University of Iowa kindly provided RAM2061 [18]. Compound purity was determined as ≥ 95% by high-performance liquid chromatography and verified by nuclear magnetic resonance [18]. Bortezomib was purchased from Millipore sigma (Burlington, MA, USA) and carfilzomib was purchased from Amgen (Thousand Oaks, CA, USA).

### Cell culture

MM.1S and RPMI-8226 cells were obtained from American Type Culture Collection (Manassas, VA, USA). JJN3 cells were obtained from DSMZ-German Collection of Microorganisms and Cell Cultures. ALMC-2 cells [26] were obtained from Dr. Diane Jelinek, Mayo Clinic (Rochester, MN). Cells were grown in media supplemented with 10% heat-inactivated fetal bovine serum (20% for JJN3), glutamine, and penicillin–streptomycin at 37 °C and 5% CO_2_. Mycoplasma testing was performed using MycoAlert mycoplasma detection kit (Lonza, Rockland, ME, USA).

### MTT

Cells were plated (3.5 × 10^4^ cells/150 μL per well) in 96-well plates and incubated in the presence or absence of drug. The MTT assay was performed as previously described [27]. Data were normalized so that the absorbance acquired from cells treated with solvent only was defined as having an MTT activity of 100%.

### ELISA

A human lambda ELISA kit (Bethyl Laboratories, Montgomery, TX, USA) was used to quantify intracellular monoclonal protein levels from the cell culture studies (ALMC-2, RPMI-8226 and MM.1S) as well as the levels of human lambda light chain in the blood samples from the xenograft study. Intracellular kappa light chain levels were detected in JJN3 cells using a human kappa ELISA kit (Bethyl Laboratories).

### Detection of apoptosis by flow cytometry

Cells were incubated for 48 h in the presence of RAM2061. PI (bortezomib or carfilzomib) was added either concurrently or sequentially (24 h after RAM2061). Cells were washed with PBS and stained with APC-conjugated Annexin V antibody and propidium iodide per the manufacturer’s instruction (eBioscience, San Diego, CA, USA). Ten-thousand cell events were recorded using a BD LSRII flow cytometer. FlowJo software was used for all data analysis. We define early apoptotic cells as being Annexin V+/Propidium iodide− and late apoptotic as Annexin V+/Propidium iodide+.

### Detection of cell surface calreticulin

Cells were incubated for 48 h in the presence of RAM2061. PI (bortezomib or carfilzomib) was added either concurrently or sequentially (24 h after RAM2061). Cells were washed with PBS and incubated with calreticulin polyclonal antibody (1:200, Invitrogen PA3-900), followed by secondary anti-rabbit Alexafluor594. Ten-thousand cell events were recorded using a BD LSRII flow cytometer. FlowJo software was used for all data analysis.

### Immunoblot

Cells were incubated for the specified amount of time in the presence or absence of RAM2061, bortezomib or carfilzomib. Cells were washed with phosphate-buffered saline (PBS) and lysed in RIPA buffer (1% sodium deoxycholate, 0.15 M NaCl, 0.1% SDS, 1% (v/v) Triton X-100, 0.05 M Tris HCl, pH 7.4) supplemented with protease and phosphatase inhibitors. Protein concentration was determined using the BCA method. Protein (15 μg) was run on an SDS-PAGE gel and transferred to a polyvinylidene difluoride membrane. Blots were incubated overnight in primary antibody and for 1 h in secondary antibody. Protein was visualized using a chemiluminescence detection kit and a Bio-Rad Chemidoc MP imaging system. Antibodies used for these assays can be found in the Additional file 1: Table S1.

### Quantitative real-time polymerase chain reaction

Cells were incubated for the specified amount of time in the presence or absence of RAM2061, bortezomib or carfilzomib. RNA was isolated using the E.Z.N.A HP total RNA kit from Omega Biotek (Norcross, GA, USA). RNA (1 μg) was reverse-transcribed to cDNA using the i-Script cDNA synthesis kit (Bio-Rad, Hercules, CA) and mixed with gene-specific primers and i-Taq Sybr green super mix (Bio-Rad) according to manufacturer’s instruction. qRT-PCR reactions were performed in triplicate using a Bio-Rad CFX96 instrument. Data analysis was performed using the Bio-Rad CFX manager 3.1 software. Gene expression was normalized to that of β-actin. Primer sequences used in these studies can be found in Additional file 1: Table S2.

### Myeloma xenografts

Female NOD/SCID mice (Charles River) between the ages of 6–8 weeks were housed in the University of Nebraska Medical Center (UNMC) animal facility at a temperature of 23–25 ℃, relative humidity of 50–70% and 12/12-h light/dark cycles. The UNMC IACUC (protocol number 16-132-11-FC) approved all animal studies. NOD-SCID mice were subcutaneously inoculated in the flank with MM.1S cells (10 million cells in 0.1 mL of sterile saline mixed 1:1 with Matrigel). When tumors became palpable (approximately 2 weeks), mice were divided into control and treatment groups to provide an equal distribution of tumor size for each group at the start of treatment (n = 8). Our pre-established practice is to exclude mice that do not have palpable tumors by the start of treatment. Thus, one mouse from both the control and RAM2061 group were excluded due to lack of tumor engraftment. Controls were administered 100 µL PBS 2×/wk (IV). Bortezomib was administered subcutaneously at a dose of 0.3 mg/kg 2×/wk. RAM2061 was administered IV at a dose of 0.08 mg/kg 2×/wk. All mice began treatment on day 14 post MM cell inoculation. Tumor volume was recorded three times per week using a caliper. Investigators were not blinded during the course of this experiment. The following equation was used to calculate tumor volume: 4π/3 × (width/2)^2 × (length/2).

### Blood analysis

Blood samples were obtained postmortem from the heart and analyzed using an Abaxis Vetscan2 instrument and the preventive profile plus rotor (Allied Analytic, Tampa, FL).

### Histology

Mouse organs were fixed in formalin for 24–96 h, stored in 70% ethanol, embedded in paraffin, sectioned, and stained (H&E or with anti-cleaved caspase 3 antibody) using standard methods at the UNMC Tissue Science Facility. To quantify the cleaved caspase 3 data, stained cells from five fields of view/sample were manually counted (ImageJ software) and averages were calculated for each sample.

### Statistics

T-tests (two-tailed) were used to calculate statistical significance between control and treated groups. Linear mixed models (LMM) were used to look at changes in tumor burden over time. Tumor burden was modeled on the log_10_ scale in order to meet model assumptions. The model included fixed effects for group, day and the group x day interaction and day is modeled as a continuous variable. A random slope and intercept were fit for each mouse. Distributional assumptions of the LMM were assessed using residual plots and the linearity and equal variance assumptions were found to be met when data was analyzed on the log_10_ scale. Kaplan–Meier method was used to estimate overall survival distributions by treatment group. Overall survival was defined as time from start of treatment to death. A log rank test with was used to calculate statistical significance of the Kaplan–Meier survival curve and the Holm-Sidak method was applied to adjust for multiple comparisons. GraphPad Prism version 7.04 (San Diego, CA, USA) and SAS software version 9.4 (Cary, NC, USA) were used for data analysis. With n = 8 mice per group we achieve 80% power to detect a mean difference in tumor burden of 200 mm^3^ with a SD = 100 mm^3^ and alpha = 0.008 (Bonferroni adjusted for 6 pairwise comparisons) using a two-sided two-sample equal-variance t-test.

## Results

### Effects of combination therapy on MM cell death

As an initial evaluation of the cytotoxic effects of combining the GGSI RAM2061 with the PI bortezomib in MM cells, MTT assays were conducted in four human MM cell lines (MM.1S, RPMI-8226, JJN3, and ALMC-2). RAM2061 was added at the beginning of the 48-h incubation period while bortezomib was added at either the same time (concurrent) or after 24 h (sequential). Isobologram analysis of the MTT data was performed and combination indices (CI) were determined (Tables 1 and 2). These studies revealed that sequential treatment of bortezomib with RAM2061 resulted in a primarily synergistic interaction (CI < 1) while concurrent treatment was primarily additive (CI = 1) to antagonistic (CI > 1) (Additional file 1: Fig. S1). Additional MTT assays were performed using the PI carfilzomib. We also observed primary synergistic (Cl < 1) to additive (CI = 1) interactions following sequential treatment of carfilzomib with RAM2061, and a primarily additive (CI = 1) to antagonistic (CI > 1) interaction when using concurrent carfilzomib and RAM2061 (Additional file 1: Fig. S2, Table S3).

**Table 1:** Summary of combination indices from MTT cytotoxicity studies for sequential bortezomib and RAM2061 treatment

Fa	ALMC-2	JJN3	MM.1S	RPMI
0.3	0.51	0.94	0.97	0.14
0.5	0.34	0.94	0.87	0.06
0.75	0.2	0.96	0.77	0.03

Cells were incubated with varying concentrations of RAM2061 and/or bortezomib. RAM2061 was added at the beginning of the 48-h incubation period. Studies were performed in which bortezomib was added 24 h after RAM2061 (sequential). Isobologram analysis was performed, and combination indices were determined. Combination indices < 1 are considered synergistic in nature

**Table 2:** Summary of combination indices from MTT cytotoxicity studies for concurrent bortezomib and RAM2061 treatment

Fa	ALMC-2	JJN3	MM.1S	RPMI
0.3	1.81	1.22	1.45	1.36
0.5	1.95	1.25	1.5	1.37
0.75	2.2	1.35	1.58	1.38

Cells were incubated with varying concentrations of RAM2061 and/or bortezomib. RAM2061 and bortezomib were added at the beginning of the 48-h incubation period (concurrent). Isobologram analysis was performed, and combination indices were determined. Combination indices > 1 are considered antagonistic in nature

**Fig. 1:**
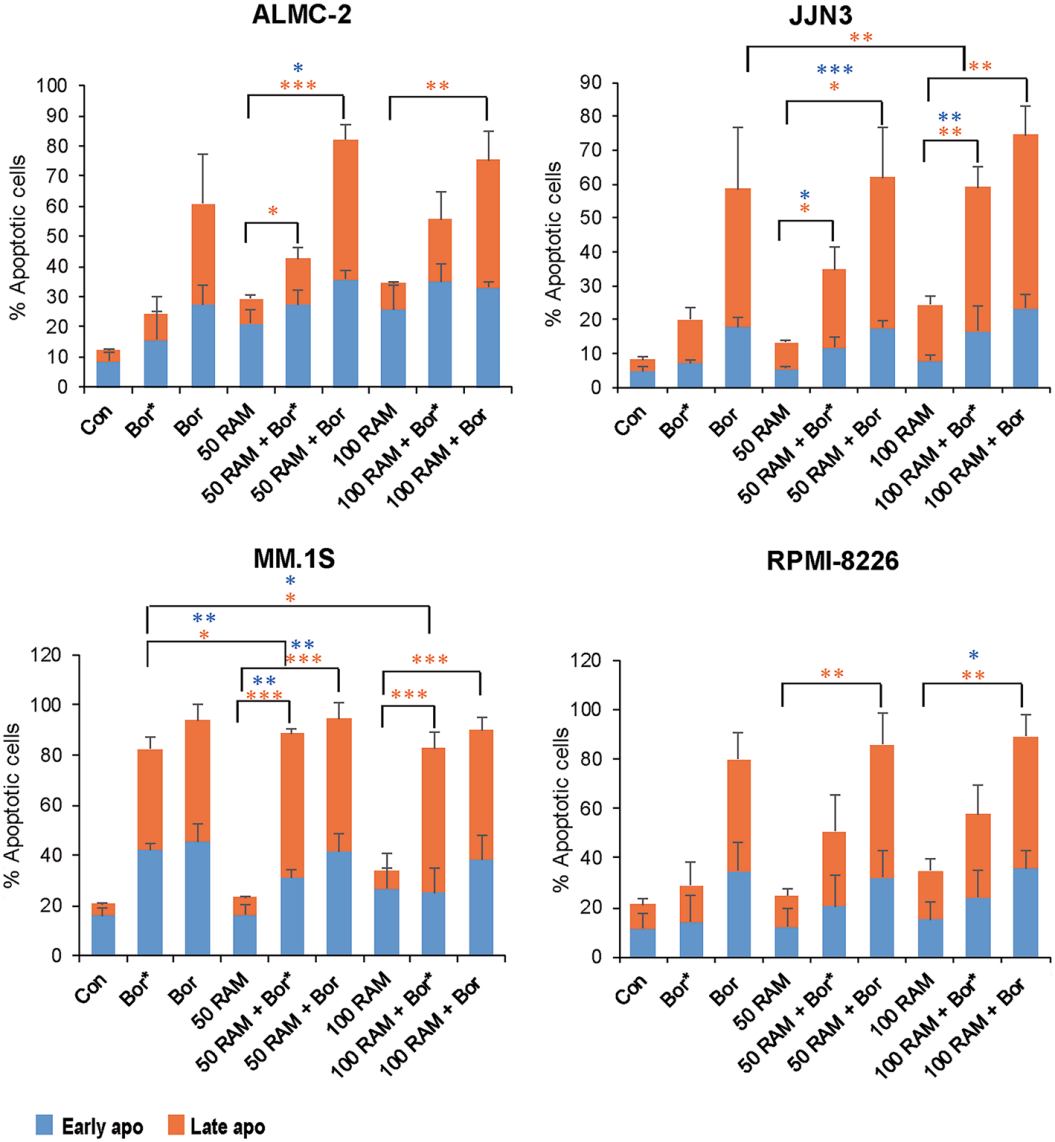
Effects of sequential and concurrent bortezomib therapy in combination with RAM2061 on induction of apoptosis. MM cells were incubated with RAM2061 (50 or 100 nM) for 48 h. Bortezomib was added either concurrently or after 24 h (sequential; denoted by *) (Bor: 3 nM (MM.1S), 5 nM (JJN3 and RPMI-8226), 15 nM (ALMC-2)). Cells were stained with fluorescently conjugated Annexin V and propidium iodide (PI) and analyzed by flow cytometry. Data are expressed as the average percentage of Annexin V+/PI− (early apoptotic) and Annexin V+/PI+ (late apoptotic) (n = 3, data are displayed as mean ± stdev, * denotes p < 0.05. ** denotes p < 0.01. *** denotes p < 0.001 per t-test)

**Fig. 2:**
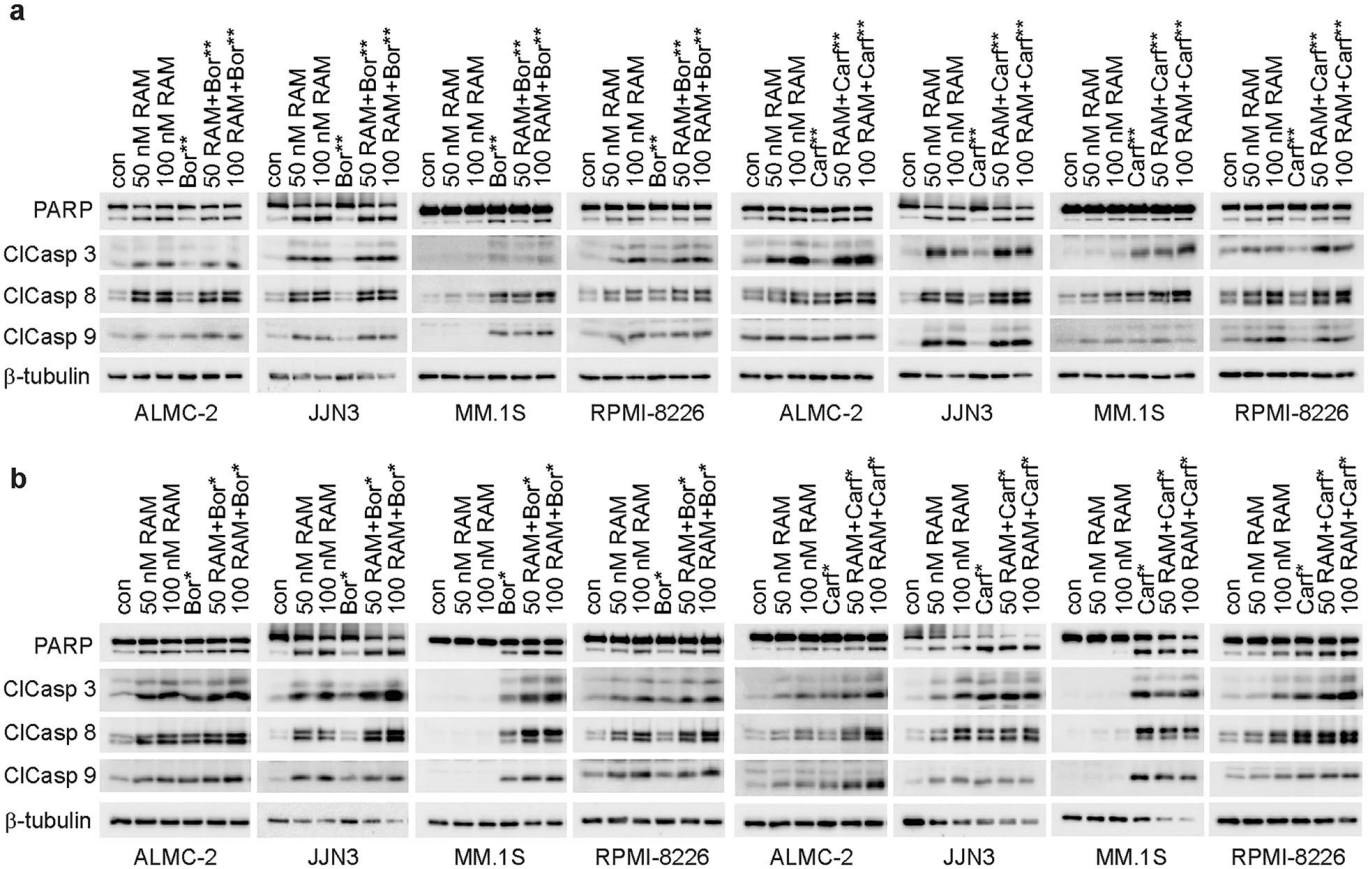
Effects of sequential PI therapy in combination with RAM2061 on markers of apoptosis. MM cells were incubated with RAM2061 (50 or 100 nM) for 48 h with or without sequential PI treatment. Bortezomib or carfilzomib was added either **a** 6 h (denoted by **) or **b** 24 h (denoted by *) prior to cell harvest (Bor: 3 nM (MM.1S), 5 nM (JJN3 and RPMI-8226), 15 nM (ALMC-2), Carf: 20 nM). Immunoblot analysis for PARP (lower band = cleaved PARP), cleaved caspases 3, 8, and 9 (ClCasp 3, 8, 9) is shown. β-tubulin was used as a loading control. Blots are representative of three independent experiments

To explore further the impact of timing of PI administration on induction of cell death with RAM2061, we assessed induction of apoptosis by flow cytometry using Annexin V and propidium iodide staining. Relative to single treatment controls, both concurrent and sequential treatment of bortezomib with RAM2061 lead to an increase in the percent of apoptotic cells (Fig. 1). Conversely, we did not observe a similar increase in apoptotic cells in cell lines treated with RAM2061 in combination with another PI, carfilzomib (Additional file 1: Fig. S3). We also examined induction of apoptosis via immunoblot analysis of cleaved PARP, caspases 3, 8, and 9. MM cells were incubated in the presence or absence of RAM2061 (50 or 100 nM) for 48 h. PI was added concurrent with RAM2061 or either 6 h (denoted as **) or 24 h (denoted as *) prior to cell harvest. We observed increased PARP and caspase cleavage in MM cells treated with RAM2061 and 6-h sequential treatment of either bortezomib or carfilzomib when compared to single drug treatment (Fig. 2a). Likewise, MM cells treated with RAM2061 and 24-h sequential bortezomib or carfilzomib displayed enhanced apoptotic markers relative to single treatment controls (Fig. 2b). Conversely, concurrent treatment with PI resulted in more rapid cell death and a consequent decrease in protein levels was observed (Additional file 1: Fig. S4).

### Comparison of UPR induction with concurrent vs sequential PI therapy in combination with RAM2061

**Fig. 3:**
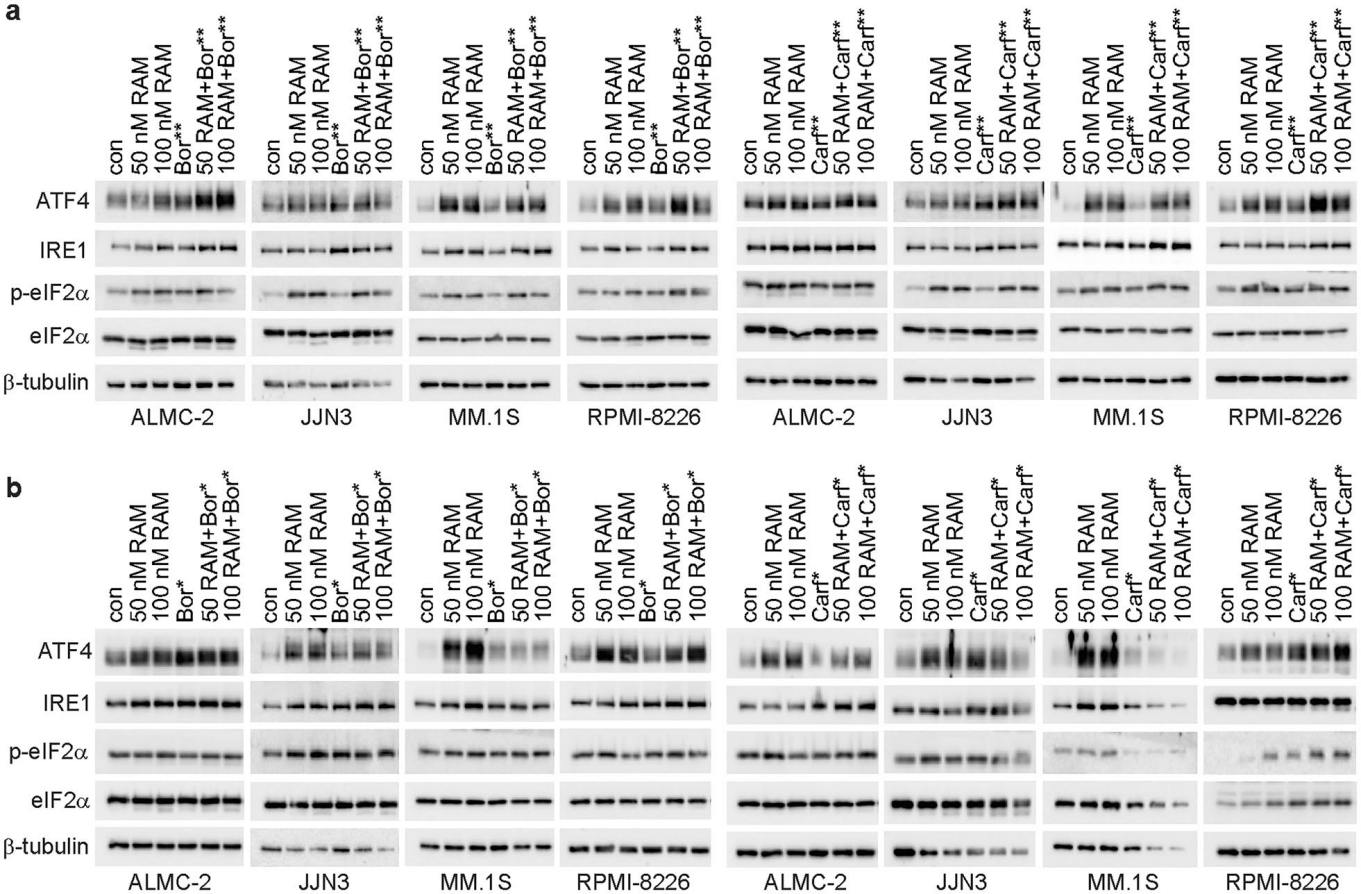
Effects of sequential PI therapy in combination with RAM2061 on markers of the unfolded protein response. MM cells were incubated with RAM2061 (50 or 100 nM) for 48 h with or without sequential PI treatment. Bortezomib or carfilzomib was added either **a** 6 h (denoted by **) or **b** 24 h (denoted by *) prior to cell harvest (Bor: 3 nM (MM.1S), 5 nM (JJN3 and RPMI-8226), 15 nM (ALMC-2), Carf: 20 nM). Immunoblot analysis for ATF4, IRE1, p-eIF2α, and eIF2α was performed. β-tubulin is shown as a loading control. Blots are representative of three independent experiments

Next, we examined markers of the UPR (ATF4, IRE1 and phosphorylated-eIF2α (p-eIF2 α)) using the same sequential and concurrent PI treatment as previously described. These studies revealed that administration of either bortezomib or carfilzomib during the final 6 h or final 24 h of incubation enhanced the RAM2061-induced upregulation of UPR markers in a cell line-specific manner (Fig. 3a and b). Concurrent 48-h treatment with PI led to rapid apoptosis and a subsequent decrease in UPR markers, likely due to the rapid effects of PI on the UPR pathway (Additional file 1: Fig. S5). qRT-PCR analysis of UPR markers revealed a partial and cell line-dependent increase in *ATF4, AT6, CHOP, IRE1 and PERK* expression levels following 6- and 24-h incubation of PI with RAM2061 relative to single treatment controls (Additional file 1: Fig. S6 and S7). Neither bortezomib nor carfilzomib enhanced RAM2061-mediated intracellular light chain retention as measured by ELISA (Additional file 1: Fig. S8).

### Combination treatment with RAM2061 and PI leads to induction of the ICD pathway

**Fig. 4:**
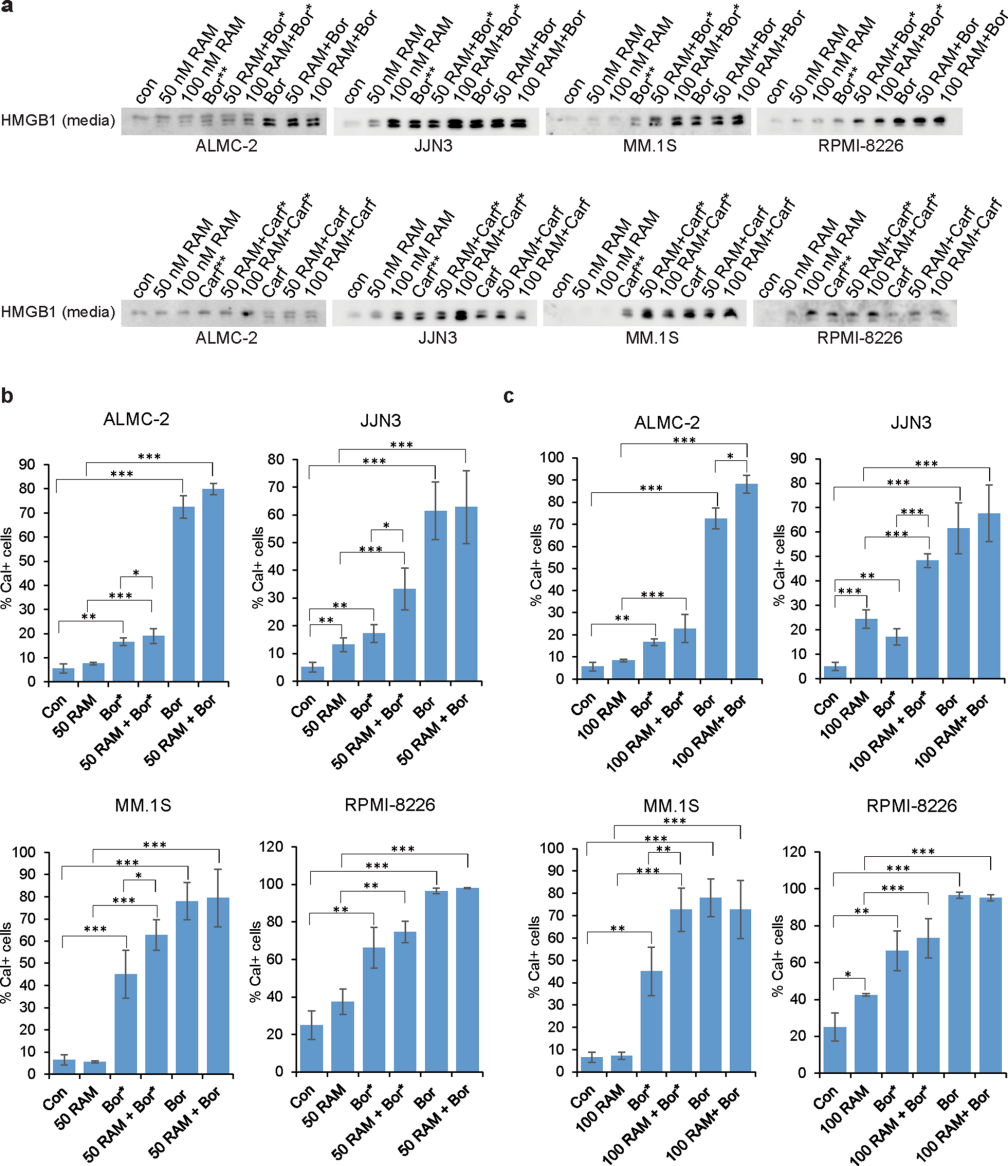
Combination treatment with RAM2061 and PI leads to induction of the ICD pathway markers. MM cells were incubated with RAM2061 (50 or 100 nM) for 48 h with or without sequential (added after 24 h, denoted by *) or concurrent PI treatment (Bor: 3 nM (MM.1S), 5 nM (JJN3 and RPMI-8226), 15 nM (ALMC-2), Carf: 20 nM). **a** Immunoblot analysis of secreted HMGB1 was performed using media collected from cells. **b**, **c** Cell surface levels of calreticulin were measured by flow cytometry. Data are expressed as the average percentage of calreticulin positive (n = 3, data are displayed as mean ± stdev, * denotes p < 0.05. ** denotes p < 0.01. *** denotes p < 0.001 per t-test). Blots are representative of three independent experiments

Next, we sought to determine if GGSI treatment, either alone or in combination with a PI, could induce the immunogenic cell death (ICD) pathway. This pathway is characterized by cell-surface translocation of calreticulin, release of damage-associated molecular pattern (DAMP) molecules (including HMGB1 (high mobility group protein B1)) and stimulation of type I interferon (IFN) responses. To measure HMBGI release, we performed immunoblot analysis on media collected from MM cells incubated with RAM2061 and PI. Both 24- and 48-h PI treatment in the presence of RAM2061 enhanced the release of HMGB1 into the media (Fig. 4a). Bortezomib induced cell-surface translocation of calreticulin following both 24- and 48-h incubation in all four cell lines, while carfilzomib and RAM2061 had little effect on cell surface levels of calreticulin when used as single agents (Fig. 4b, c and Additional file 1: Fig. S9). Both concurrent and sequential incubation of bortezomib in the presence of RAM2061 enhanced cell surface translocation of calreticulin relative to single drug treatments (Fig. 4b, c).

### Effects of RAM2061 and/or bortezomib treatment in MM mouse xenografts

**Fig. 5:**
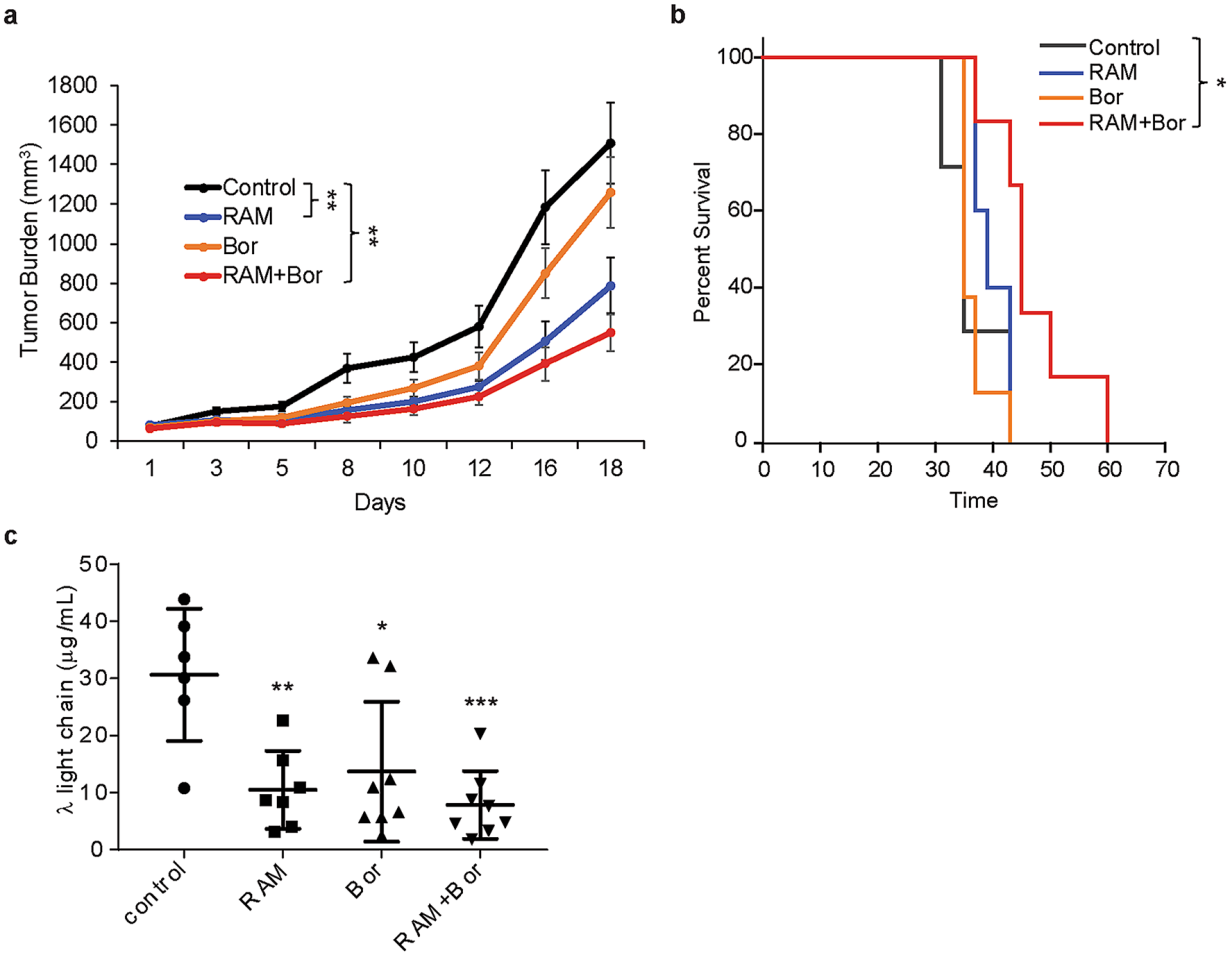
Effects of RAM2061 and/or bortezomib treatment in MM mouse xenografts. **a** Tumor growth curves for MM.1S flank xenografts. (*n* = 8 mice per group, data are displayed as mean ± SEM, denotes *p* < 0.01 per mixed-linear mixed model). **b** Kaplan–Meier curve for MM.1S flank xenografts showing time to sacrifice. Mice were treated twice-weekly with either solvent only control (PBS), RAM2061, Bort, or a combination of RAM2061 and Bort. Mice were euthanized when tumors reached 2000 mm^3^. * denotes p < 0.05 per long rank test. **c** Levels of human λ light chain blood samples drawn on day 9 post-initiation of treatment. * denotes p < 0.05, **p < 0.01 and ***p < 0.001 per t-test

Using a MM flank xenograft model, we examined if combination treatment of bortezomib with RAM2061 would be efficacious in slowing tumor growth in vivo. NOD-SCID mice were inoculated with MM.1S cells in the flank and treated with PBS (control), bortezomib, RAM2061, or a combination of RAM2061/bortezomib. Tumor growth over time was significantly decreased in the RAM2061 (p = 0.0009) and in the RAM2061/bortezomib (p = 0.0002) treatment groups compared to control animals (Fig. 5a). In addition, survival, or time to sacrifice, was significantly prolonged in the RAM2061/bortezomib cohort (p = 0.003), but not the RAM2061 group (p = 0.09, Fig. 5b). Concordant with tumor volume, mean plasma human lambda light chain levels were decreased in the RAM2061 and to a larger degree in the RAM2061/bortezomib treatment groups when compared to the control mice (p < 0.05, Fig. 5c). Consistent with our previous in vivo studies, the primary toxicity associated with RAM2061 treatment was hepatic transaminase elevation (Additional file 1: Table S4). Histological examination of livers revealed mild and variable lobular unrest (defined as variable nuclear enlargement with a rare binucleated hepatocyte) around the central vein (zone 3) likely related to mild regenerative changes in bortezomib treated mice (Additional file 1: Fig. S10). Livers of RAM2061-treated mice displayed prominent lobular unrest around zone 3 with rare apoptotic hepatocytes and occasional lipofuscin-laden macrophages which signify hepatocyte dropout. Livers of mice treated with both drugs showed the same prominent lobular in zone 3 with adjacent ballooning degeneration in zone 2 hepatocytes. Spleen and kidney morphology were normal across all groups (Additional file 1: Fig. S10). No additional toxicity was observed when bortezomib was combined with RAM2061 based on animal weight, renal or hepatic parameters (Additional file 1: Fig. S11 and Table S4). We observed a trend toward greater cleaved caspase 3 expression in tumors isolated from mice treated with RAM2061 relative to controls (Additional file 1: Fig. S12).

## Discussion

RAM2061 is a homoneryl triazole bisphosphate that functions as a potent GGDPS inhibitor with an IC_50_ of 86 nM and lowest effective concentration in MM cells of 25 nM [18]. Our preclinical studies demonstrate that RAM2061 displays a prolonged terminal half-life of 29.2 h, metabolic stability, in vivo on target effects, and systemic distribution including favorable bone marrow exposure [24]. In addition, single agent activity was observed in a xenograft model using human MM.1S cells. Notably, this is the first study to show that RAM2061 and bortezomib have synergistic cytotoxic activity in MM cells. Further investigation revealed that bortezomib and carfilzomib are able to potentiate induction of markers of the UPR and ICD pathways in the presence of RAM2061. In addition, we present the first in vivo investigation showing the anti-tumor effects of combination GGSI/PI inhibitor therapy in a xenograft tumor model.

The proteasome is tasked with degrading the majority of cellular proteins and thus plays a pivotal role in maintaining protein homeostasis. PIs induce MM cell death via various well-documented mechanisms, including the inhibition of several pro-survival factors, such cytokines, adhesion molecules, angiogenesis, and NF-κB [6–8, 28]. In addition, PI-mediated accumulation of misfolded protein and induction of the UPR in MM cells has been described by several accounts in the literature. One study showed that bortezomib induced the accumulation of misfolded ER-processed proteins, which led to activation of the pro-apoptotic arm of the UPR, and that MM cells sensitivity to bortezomib directly correlates with the amount of MP retained in the cell [1]. In another study, a specific inhibitor of the proteasome β2 subunit activated the UPR and induced cytotoxicity in bortezomib-resistant MM cells when combined with either bortezomib or carfilzomib [29]. Dual inhibition of the β2 and β5 subunits using a PI termed syringolog-1 resulted in increased expression of CHOP, ATF3, and XBP1, as well as induction of apoptosis in both bortezomib resistant and sensitive cell lines [30]. Likewise, we observed upregulation of several UPR mediators upon bortezomib or carfilzomib treatment, including ATF4, IRE1, and p-eIF2a. Our current and previous studies have demonstrated that GGSIs induce all three arms of the UPR [23–25]. While our current studies showed several UPR markers that were further upregulated following combination therapy, the underlying mechanism does not appear to be a direct consequence of accumulation of intracellular MP as the addition of PI did not enhance the effects of the GGSI (Additional file 1: Fig. S8). Prior work has demonstrated that PIs can induce utilization of non-proteasome-mediated degradation pathways such as aggresomes and autophagosomes, thus alleviating the ER stress [31–34]. However, our prior studies with IBP inhibitors demonstrated that these agents do not increase aggresome numbers in MM cells and that that the accumulated intracellular MP induced by these inhibitors is not a substrate for aggresomes, autophagosomes or lysosomes [35]. Notably, the effect of PIs on augmenting RAM2061-induced upregulation of UPR markers was both concentration- and time-dependent. Concurrent exposure results in less induction of the UPR pathway, likely as a result of the more rapid effects of bortezomib on this pathway relative to RAM2061, with subsequent decrease in markers as cells undergo apoptosis. These results likely contribute to the effects observed in the MTT assays, where concurrent treatment appeared to induce less robust effects on cytotoxicity (as indicated by CI’s showing additive/antagonistic interactions) than sequential treatment strategies. Further studies are needed to dissect the mechanisms underlying the interactions between the two classes of drugs on the UPR and apoptotic pathways and to explore alternative dosing schedules that would maximize in vivo efficacy.

There is rationale in the literature to suggest that use of IBP pathway inhibitors would be of therapeutic value as anti-cancer agents. Two studies using the GGDPS inhibitor GGOHBP showed in vivo reduction in tumor burden in both adrenal and prostate cancer xenografts [36, 37]. Another preclinical study using a series of thienopyrimidine-based GGDPS inhibitors and the a Vk*MYC transgenic mouse model demonstrated that GGDPS inhibition decreased serum MP levels [38]. Our own studies with RAM2061 or a similar structural analog (VSW1198), show single agent activity in both MM.1S flank xenografts as well as pancreatic cancer xenografts [24, 39]. To date, there is one account in the literature that evaluates the benefit of combining IBP pathway inhibitors with PI therapy. This study shows that statins, which inhibit HMG-CoA reductase in the mevalonate pathway and lead to downstream disruption in isoprenoid synthesis, enhanced induction of apoptosis and UPR markers when used in combination with bortezomib in *t*(4;14)-positive MM cells [40]. Furthermore, using add-back studies they demonstrate that GGPP depletion drives apoptosis and UPR activation in their model. Our present studies have demonstrated synergy between the GGSI and bortezomib in t(4;14)-negative MM cell lines that have other high-risk cytogenetic abnormalities including t(14;16) or del(17p) [26, 41].

While PIs remain the backbone of MM treatment, development of relapsed/refractory disease has led many researchers to begin testing combination PI treatment with inhibitors of lesser explored pathways. Okabe et al. showed that combined treatment with carfilzomib and CUDC-907, a dual PI3-K and histone deacetylase inhibitor, enhanced cellular toxicity in human MM cell lines when compared to single-drug treatment [42]. In another report, inhibition of phosphoglycerate dehydrogenase (PHGDH) using the inhibitor NT-503, significantly potentiated the cytotoxic effect of bortezomib in seven out of eight tested MM cell lines [43]. Furthermore, combination treatment with NCT-503 and bortezomib displayed enhanced anti-tumor effects in the 5T33MM mouse model when compared to single drug treatments. Together, these studies along with our own demonstrate the therapeutic potential of targeting novel pathways in conjunction with the mainstay PIs and may one day lead to the development of new treatment combinations.

Here we demonstrate that in addition to UPR activation, combination GGSI and PI enhanced the activation of the ICD pathway. While it has been overlooked in favor of studying direct cytotoxic effects, understanding and harnessing ICD is now considered equally important in evaluating a drugs mechanism of action [44, 45]. The ICD pathway involves the release of damage-associated molecular patterns (DAMPs) by the cancer cell, which attract and activate antigen-presenting cells leading to antitumor immunity [46]. In culture, bortezomib has been shown to be capable of inducing ICD and antitumor immunity by initiating the uptake of Hsp90-expressing human MM cells by dendritic cells [47]. In our studies, bortezomib was the most potent inducer of ICD markers (calreticulin and HMGB1) as a single agent. There are no accounts in the literature describing either carfilzomib or GGSI effects in inducing ICD, however we demonstrate that both compounds can induce HMGB1 release and calreticulin translocation in a cell-line dependent manner. Future experiments dissecting the role ICD induction by PI/GGSI combination therapies plays in vivo are needed.

## Conclusion

In conclusion, our cell culture studies reveal that combination treatment with GGSI and PIs (bortezomib or carfilzomib) increases MM cell death and UPR activation and that this effect is dependent on the timing of drug exposure. Furthermore, we demonstrate that PI/GGSI treatment can potentiate upregulation of markers associated with the immunogenic cell death pathway. These studies are the first to demonstrate the feasibility of combining GGSI and PI therapy in vivo and show evidence of increased efficacy, paving the way towards future preclinical and clinical studies evaluating this novel strategy of disrupting protein homeostasis via multiple mechanisms in MM.

## Supplementary Information

**Additional file 1.** Additional figures and Tables.

## Data Availability

All data generated or analyzed during this study are included in this published article (and its Additional files). The datasets analyzed during this study are available from the corresponding author on reasonable request.
