# Supplementary material for: Geranylgeranyl diphosphate synthase inhibitor and proteasome inhibitor combination therapy in multiple myeloma
Source: Exp Hematol Oncol. 2022 Feb 9;11:5. doi: 10.1186/s40164-022-00261-6 (PMC8827146; doi:10.1186/s40164-022-00261-6)
Supplement: Supplementary file 1 — Additional file 1. Additional figures and Tables. [file 40164_2022_261_MOESM1_ESM.docx]

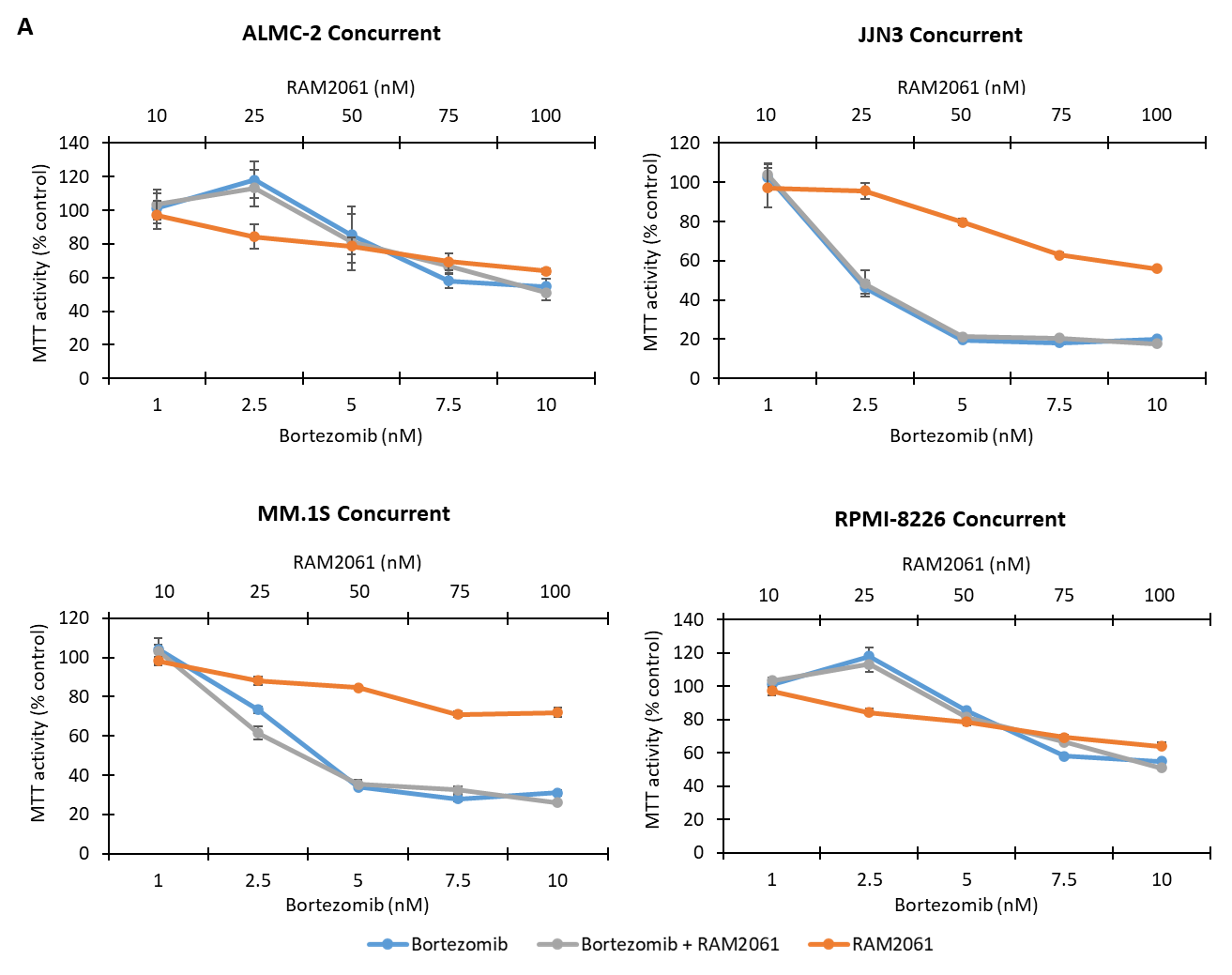


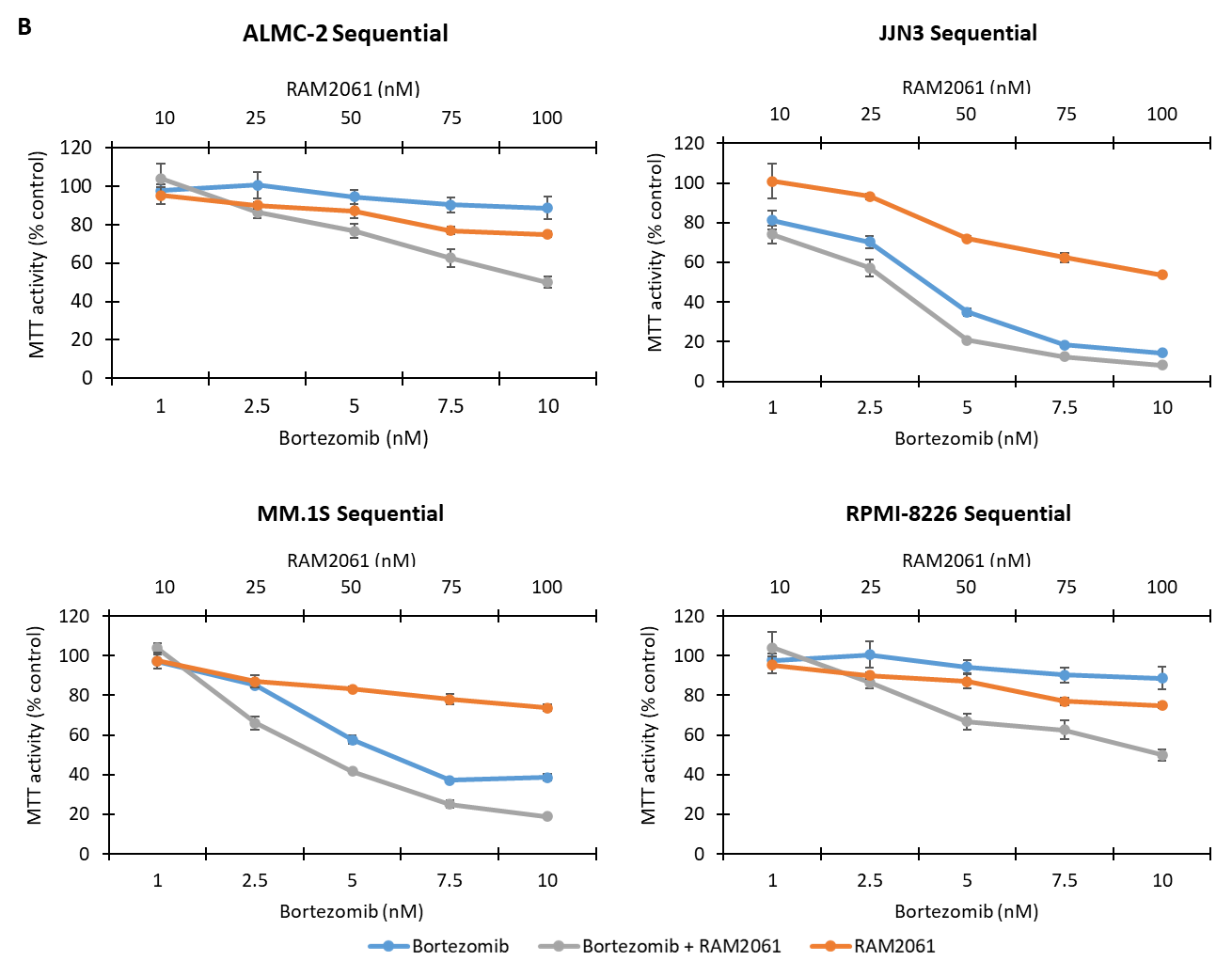


**Figure S1. The nature of the interaction between RAM2061 and bortezomib is dependent on timing of bortezomib exposure.** In the concurrent studies (A), MM cells were incubated with RAM2061 and bortezomib for 48 hours. In the sequential studies (B), MM cells were incubated with RAM2061 for 48 hours, with bortezomib added during the last 24 hours. MTT cytotoxicity assays were performed. Data are displayed as mean ± stdev (n=4).


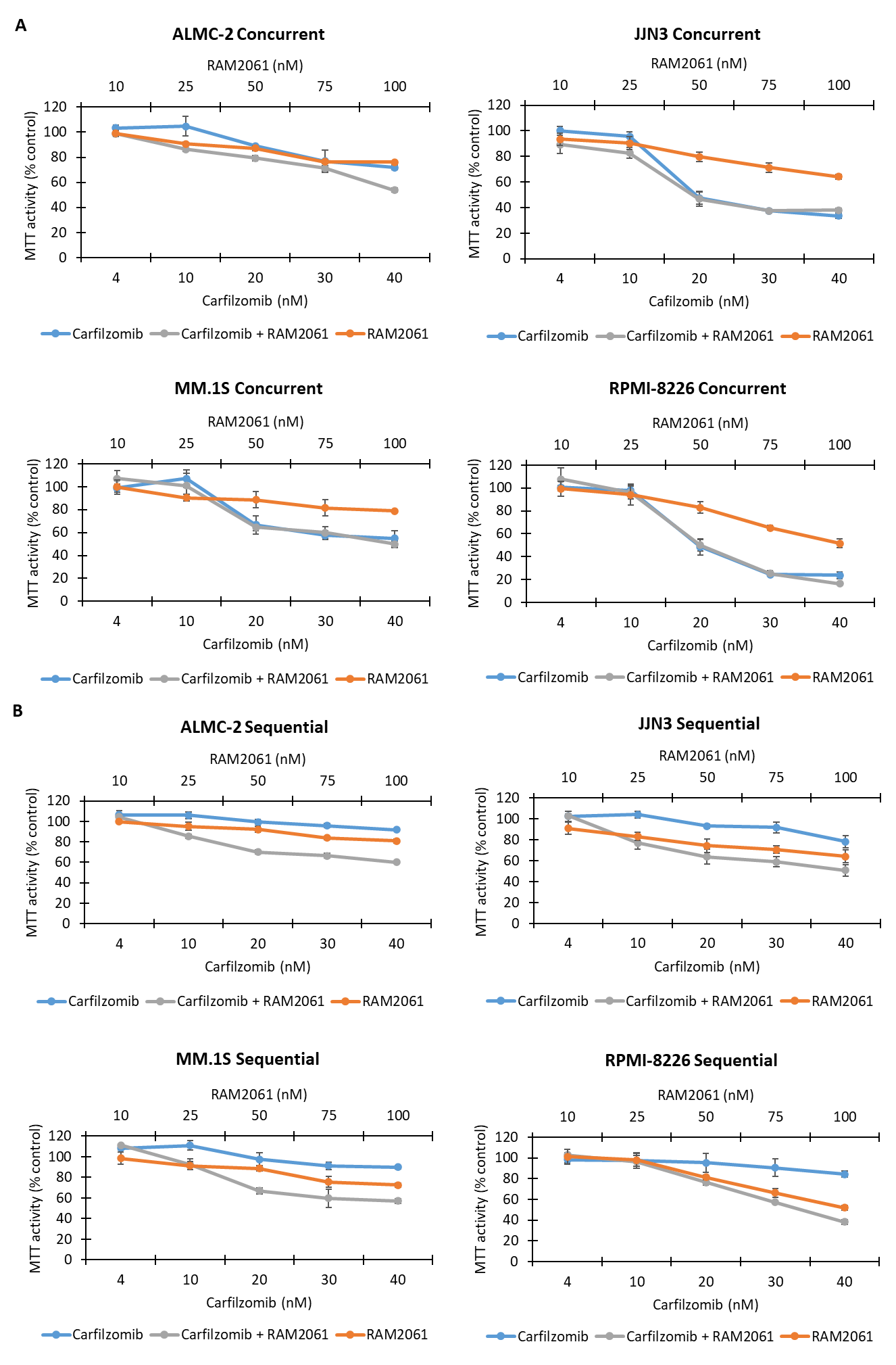


**Figure S2. The nature of the interaction between RAM2061 and carfilzomib is dependent on timing of carfilzomib exposure.** In the concurrent studies (A), MM cells were incubated with RAM2061 and carfilzomib for 48 hours. In the sequential studies (B), MM cells were incubated with RAM2061 for 48 hours, with carfilzomib added during the last 24 hours. MTT cytotoxicity assays were performed. Data are displayed as mean ± stdev (n=4).
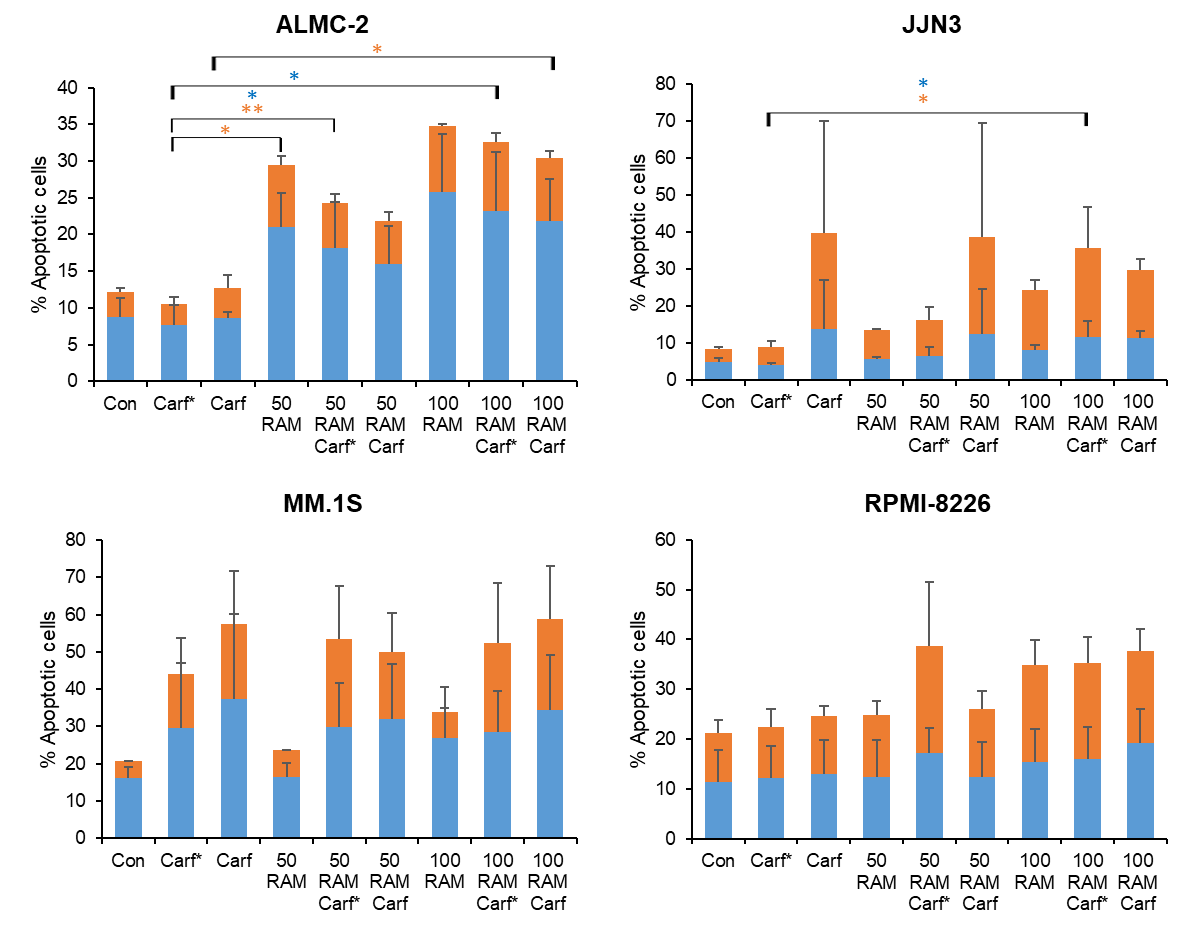


**Figure S3. Effects of sequential and concurrent carfilzomib therapy in combination with RAM2061 on induction of apoptosis.** MM cells were incubated with RAM2061 (50 or 100 nM) for 48 hours with or without sequential (added after 24 hours, denoted by *) or concurrent (added at the same time as RAM2061) carfilzomib (20 nM) treatment. Cells were stained with fluorescently conjugated Annexin V and propidium iodide (PI) and analyzed by flow cytometry. Data are expressed as the average percentage of Annexin V+/PI- (early apoptotic) and Annexin V+/PI+ (late apoptotic) (n = 3 independent experiments, data are displayed as mean ± stdev, *denotes p < 0.05. **denotes p < 0.01. ***denotes p < 0.001 per two-tailed t-test).


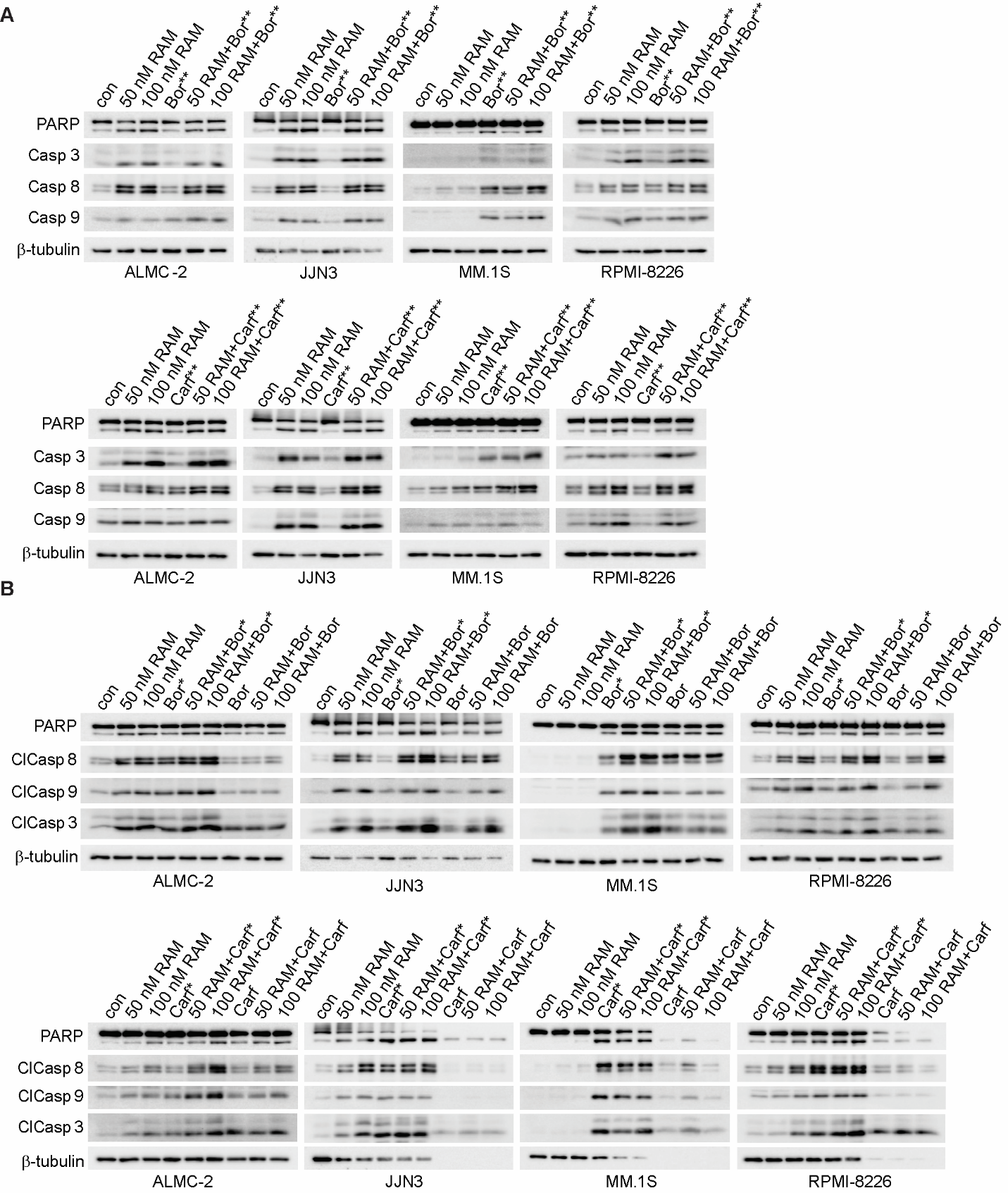


**Figure S4.** **Effects of concurrent PI therapy in combination with RAM2061 on markers of apoptosis.** MM cells were incubated with RAM2061 (50 or 100 nM) for 48 hours with or without PI treatment (Bor: 3 nM (MM.1S), 5 nM (JJN3 and RPMI-8226), 15 nM (ALMC-2); Carf: 20 nM). Bortezomib or carfilzomib was added either 24 hours (denoted by *) prior to cell harvest or concurrently with RAM2061. Immunoblot analysis for PARP (lower band = cleaved PARP), cleaved caspases 3, 8, and 9 (ClCasp 3, 8, 9) was performed. β-tubulin is shown as a loading control. Blots are representative of three independent experiments. Lanes 1-6 in each of the sets of blots are presented in Figure 1 of the main text.


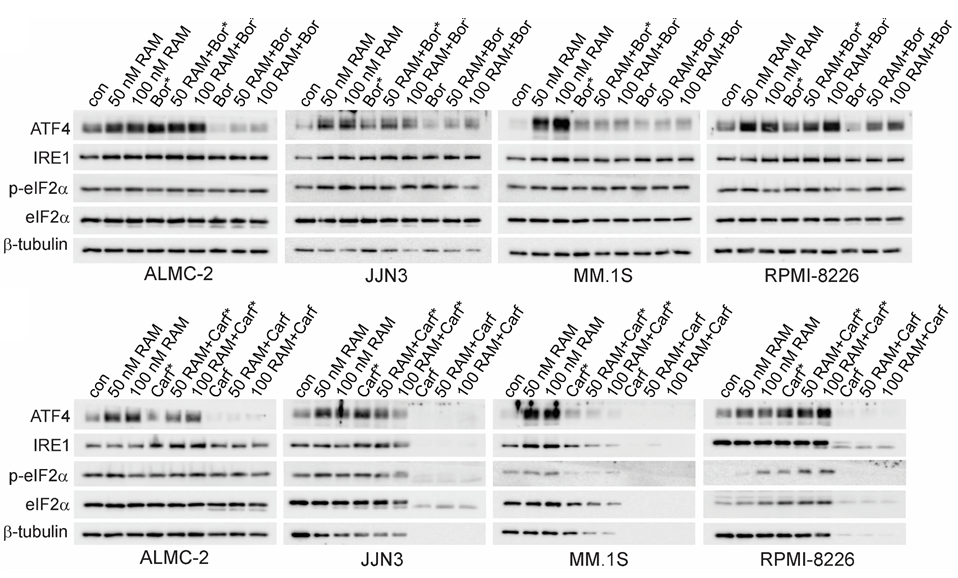


**Figure S5.** **Effects of concurrent PI therapy in combination with RAM2061 on markers of the unfolded protein response.** MM cells were incubated with RAM2061 (50 or 100 nM) for 48 hours with or without PI treatment (Bor: 3 nM (MM.1S), 5 nM (JJN3 and RPMI-8226), 15 nM (ALMC-2); Carf: 20 nM). Bortezomib or carfilzomib was added either 24 hours (denoted by *) prior to cell harvest or concurrently with PI. Immunoblot analysis for ATF4, IRE1, p-eIF2α, and eIF2α was performed. β-tubulin is shown as a loading control. Blots are representative of three independent experiments. Lanes 1-6 in each of the sets of blots are presented in Figure 3 of the main text.


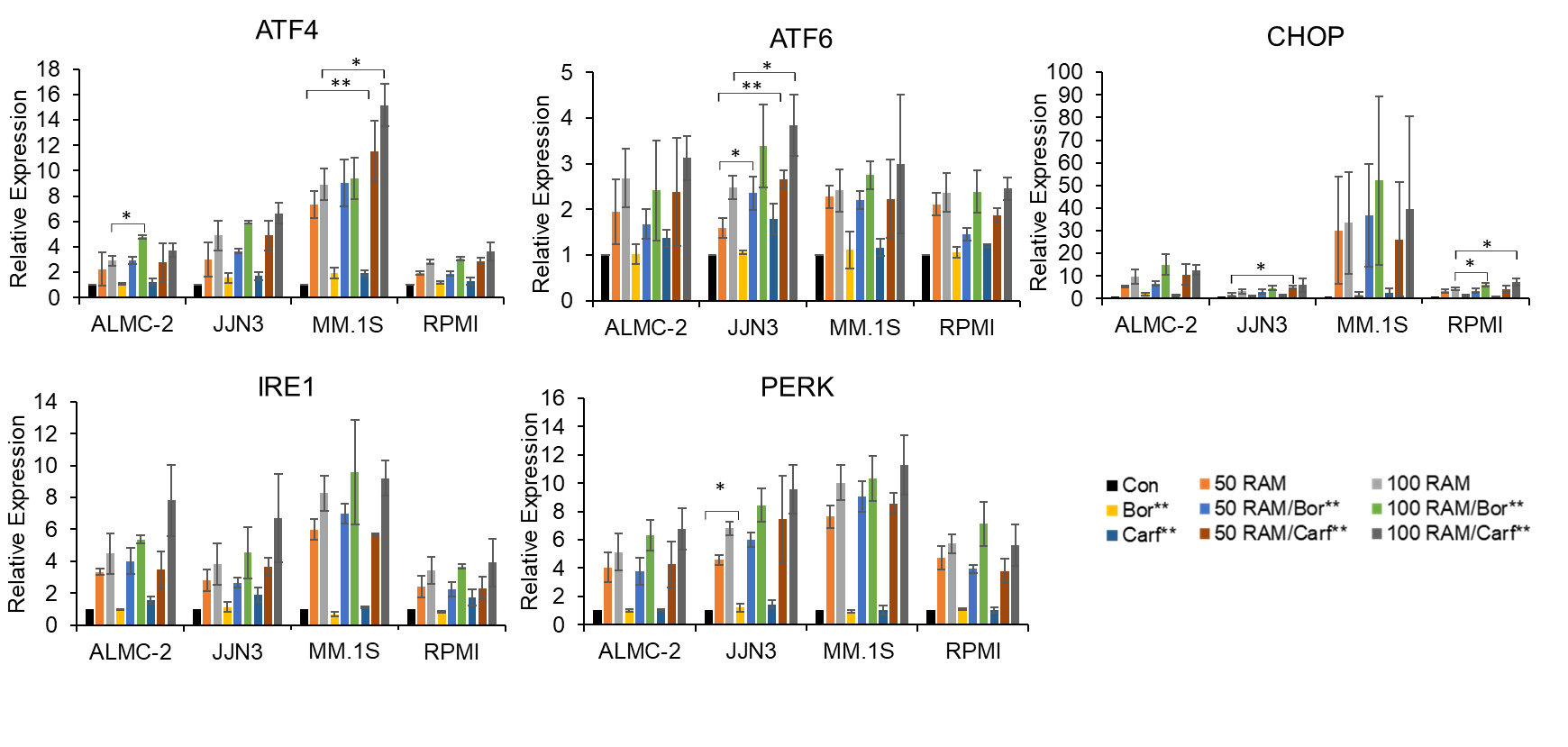


**Figure S6. Induction of UPR markers following RAM2061 and 6-hour PI co-incubation.** Cells were incubated in the presence of RAM2061 (50 or 100 nM) for 48 hours. Bortezomib (3 nM (MM.1S), 5 nM (JJN3 and RPMI-8226), 15 nM (ALMC-2)) or carfilzomib (20 nM) was added 6 hrs prior to cell harvest (Bor**/Carf**). qRT-PCR was performed. Data represents fold-change normalized to control (n=3 independent experiments). ** denotes p<0.01, *** p<0.001 per two-tailed t-test
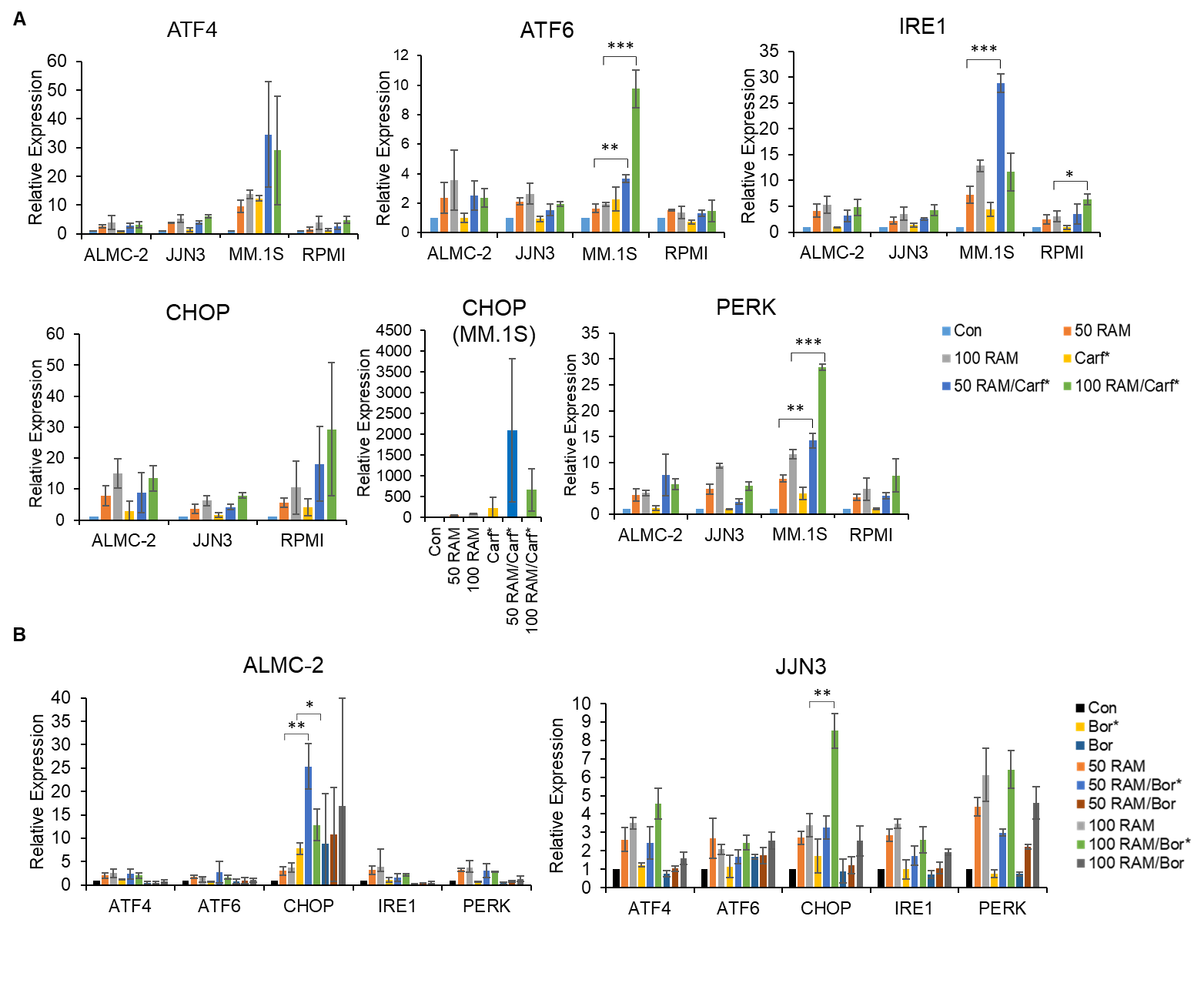
 **Figure S7. Induction of UPR markers following RAM2061 and PI co-incubation.** Cells were incubated in the presence or absence of RAM2061 (50 or 100 nM) for 48 hours. (A) Carfilzomib (20 nM) was added 24 hours prior to cell harvest (Carf*). B) Bortezomib (3 nM (MM.1S), 5 nM (JJN3 and RPMI-8226), 15 nM (ALMC-2)) was added either concurrently with RAM2061 (Bor) or 24 hours prior to cell harvest (Bor*). qRT-PCR was performed. Data represents fold change normalized to control (n=3 independent experiments). ** denotes p<0.01, *** p<0.001 per two-tailed t-test.


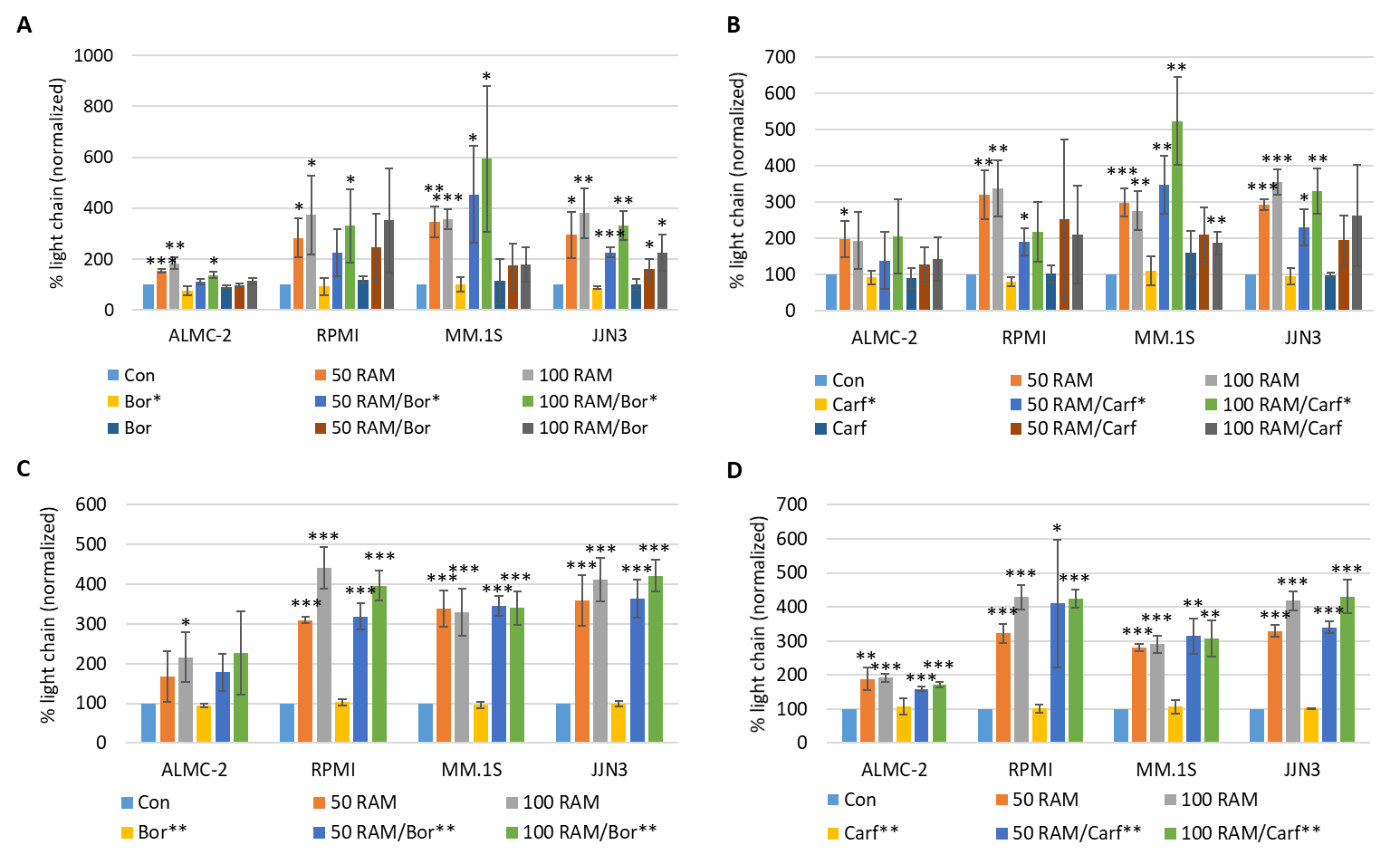


**Figure S8.** **Effects of concurrent and sequential PI therapy in combination with RAM2061 on intracellular monoclonal protein levels.** Cells were incubated in the presence or absence of RAM2061 (50 or 100 nM) for 48 hours. (A) Bortezomib (3 nM (MM.1S), 5 nM (JJN3 and RPMI-8226), 15 nM (ALMC-2)) was added either concurrently with RAM2061 (Bor) or 24 hours prior to cell harvest (Bor*). (B) Carfilzomib (20 nM) was added either concurrently with RAM2061 (Carf) or 24 hours prior to cell harvest (Carf*). (C) ) Bortezomib (3 nM (MM.1S), 5 nM (JJN3 and RPMI-8226), 15 nM (ALMC-2)) was added 6 hours prior to cell harvest (Bor**). (D) Carfilzomib (20 nM) was added 6 hours prior to cell harvest (Carf**). Intracellular lambda (ALMC-2, MM.1S, RPMI-8226) or kappa (JJN3) light chain concentrations were determined via ELISA. Data are expressed as percentage of control (mean + SD, n=3). ** denotes p<0.01, *** p<0.001 per two-tailed t-test.


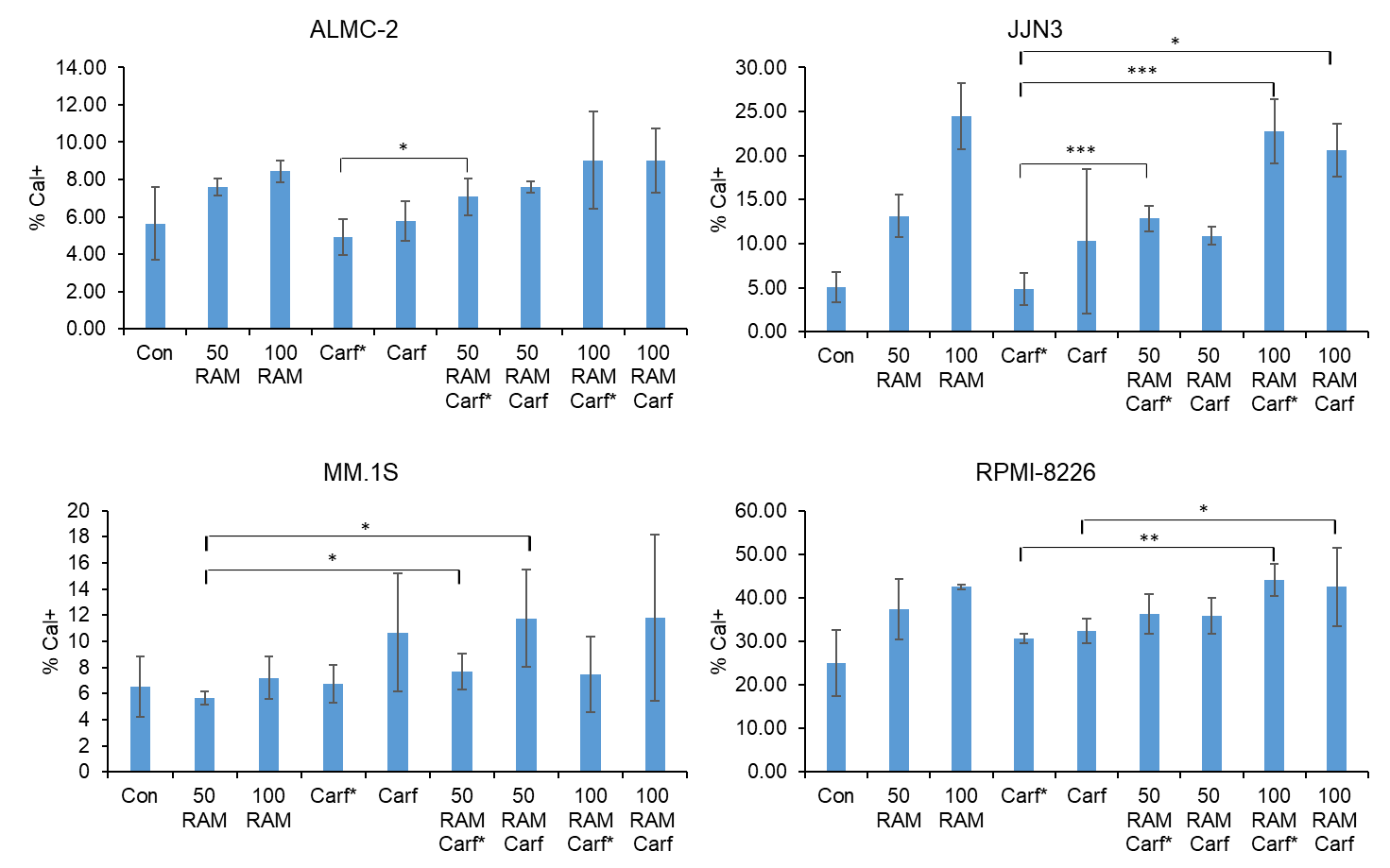


**Figure S9. Effects of combination treatment with RAM2061 and carfilzomib on calreticulin translocation to the cell surface.** MM cells were incubated with RAM2061 (50 or 100 nM) for 48 hours. Carfilzomib (20 nM) was added 24 hours prior to cell harvest (Carf*) or concurrently with RAM2061 (Carf). Cell surface levels of calreticulin were measured by flow cytometry. Data are expressed as the average percentage of calreticulin-positive cells (n = 3 independent experiments, data are displayed as mean ± stdev, *denotes p < 0.05. **denotes p < 0.01. ***denotes p < 0.001 per two-tailed t-test).


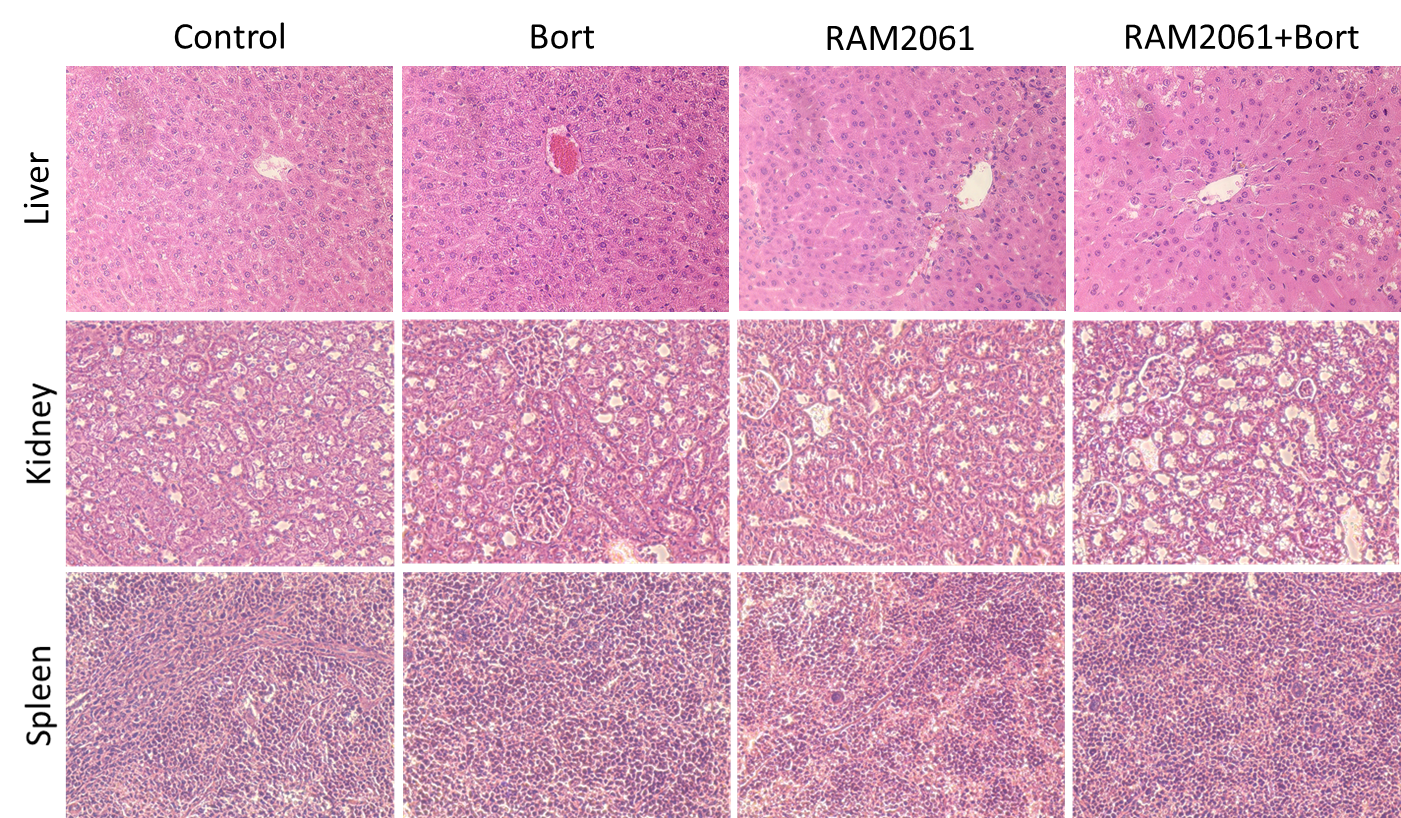


**Figure S10. Liver, kidney, and spleens collected from the MM xenograft study show no histological abnormalities.** NOD-SCID mice were inoculated with MM.1S cells in the flank and treated with PBS (control), bortezomib (Bort), RAM2061, or a combination of RAM2061 and bortezomib. Representative examples are shown. 400X magnification.


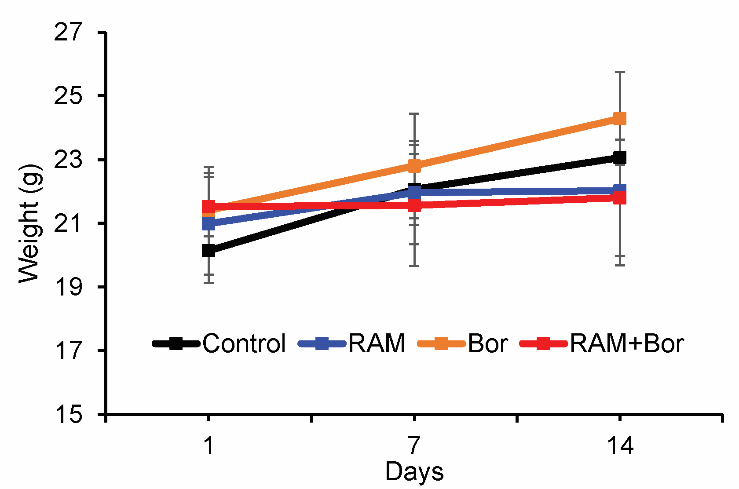


**Figure S11. RAM2061 with or without bortezomib treatment does not significantly affect animal weight.** NOD-SCID mice were inoculated with MM.1S cells in the flank and treated with PBS (control), RAM2061 (RAM), bortezomib (Bor), or a combination of RAM/Bor. Animal weight was recorded weekly. Error bars denote standard deviation (n=8 per group).


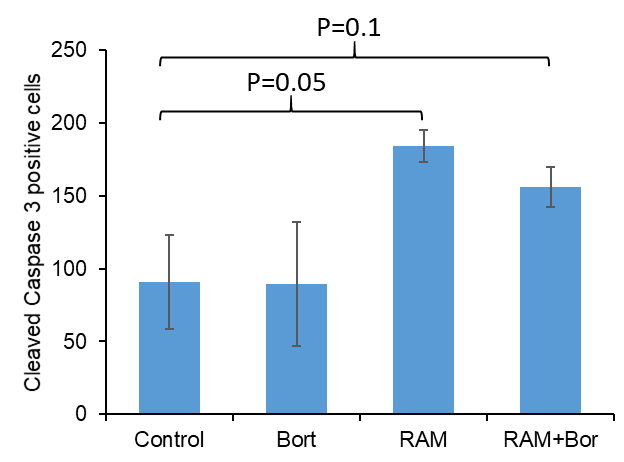


**Figure S12. Effects of RAM2061 treatment on cleaved caspase 3 expression in mouse tumors.** NOD-SCID mice were inoculated with MM.1S cells in the flank and treated with PBS (control), RAM2061 (RAM), bortezomib (Bor), or a combination of RAM/Bor. Tumors were isolated at terminal end points and tissue sections were stained using an antibody that detects cleaved caspase 3. Cleaved caspase 3-stained cells were manually counted from five fields of view from each tumor section to obtain an average (n=3 per group). Data are shown as an average across the three biological replicates. Error bars denote standard error.

| **Antibody** | **Company** | **Cat. #** | **Dilution** |
| --- | --- | --- | --- |
| ATF-4 | Cell Signaling | 11815 | 1:1000 |
| Cleaved Caspase 3 | Cell Signaling | 9664 | 1:500 |
| Cleaved Caspase 8 | Cell Signaling | 9496 | 1:500 |
| Cleaved Caspase 9 | Cell Signaling | 9501 | 1:500 |
| eIF2α | Cell Signaling | 9722 | 1:1000 |
| p-eIF2α | Cell Signaling | 3597 | 1:1000 |
| IRE1α | Cell Signaling | 3294 | 1:2000 |
| PARP | Santa Cruz | sc-7150 | 1:10,000 |
| β-Tubulin | Sigma | T5201 | 1:25,000 |
| HMGB1 | R&D | MAB1690 | 1:5000 |

**Table S1. List of antibodies used in immunoblot studies.**

| **ATF4** | |
| --- | --- |
| **F** | ACCTTCTGACCACGTTGGATGACA |
| **R** | TTCCAGGTCATCTATACCCA |
| **β-ACTIN** | |
| **F** | GGCTGTATTCCCCTCCATCG |
| **R** | CCAGTTGGTAACAATGCCATGT |
| **CHOP** | |
| **F** | ATGAGGACCTGCAAGAGGTCC |
| **R** | TCCTCCTCAGTCAGCCAAGC |
| **IRE1** | |
| **F** | AGACTTTGTCATCGGCCTTTGCAG |
| **R** | ATTCACTGTCCACAGTCACCACCA |
| **PERK** | |
| **F** | ACTCCGTGAGTGCCGATGT |
| **R** | GGAGCCGGATCCTCTTGATA |
| **ATF6** |  |
| **F** | GCCTCCACCTCCATGTCAAC |
| **R** | GAGAATGCGGGCTTTCTGTT |

**Table S2. List of primer sequences.**

**Sequential:**

| **Fa** | **ALMC-2** | **JJN3** | **MM1S** | **RPMI** |
| --- | --- | --- | --- | --- |
| 0.3 | 0.77 | 0.73 | 0.94 | 0.93 |
| 0.5 | 0.97 | 0.74 | 0.92 | 0.80 |
| 0.75 | 1.67 | 0.81 | 0.92 | 0.69 |

**Concurrent:**

| **Fa** | **ALMC-2** | **JJN3** | **MM1S** | **RPMI** |
| --- | --- | --- | --- | --- |
| 0.3 | 1.19 | 1.00 | 1.23 | 1.58 |
| 0.5 | 1.01 | 1.21 | 1.66 | 1.49 |
| 0.75 | 0.82 | 1.72 | 2.45 | 1.40 |

**Table S3. Summary of combination indices from MTT cytotoxicity studies for sequential and concurrent carfilzomib and RAM2061 treatment.** Cells were incubated with varying concentrations of RAM2061 and/or carfilzomib. RAM2061 was added at the beginning of the 48-hour incubation period. Studies were performed in which carfilzomib was added 24 hours after RAM2061 (sequential) and at the beginning of the 48-hour incubation period (concurrent). Isobologram analysis was performed, and combination indices were determined. Combination indices <1 are considered synergistic in nature, while combination indices >1 are considered antagonistic in nature.

|  | **Normal range** | **RAM2061** | **Bor** | **RAM2061+Bor** |
| --- | --- | --- | --- | --- |
| **BUN** | 9 to 33 | 20 ± 5.5 | 16.5 ± 1.7 | 18.3 ± 2.1 |
| **CRE** | 0.2 to 0.9 | 0.25 ± 0.1 | 0.3 ± 0.1 | 0.3 ± 0.1 |
| **ALT** | 17 to 77 | 461 ± 538.7 | 39 ± 8.6 | 329.5 ± 220 |
| **ALP** | 35 to 222 | 129 ± 114.4 | 37.5 ± 7.9 | 119 ± 62.2 |
| **AST** | 54 to 298 | 911.3 ± 729.9 | 145 ± 77.5 | 695.3 ± 374.7 |
| **TBIL** | 0 to 0.9 | 0.3 ± 0.1 | 0.23 ± 0.1 | 0.3 ± 0.1 |
| **GLU** | 140 to 263 | 142.8 ± 31.6 | 160.8 ± 28.9 | 155 ± 32.1 |
| **CA** | 6 to 13 | 11.1 ± 0.2 | 10.7 ± 0.7 | 11.8 ± 0.2 |
| **TP** | 3.9 to 6.4 | 4.8 ± 0.4 | 5.4 ± 0.3 | 5 ± 0.2 |
| **ALB** | 2.5 to 4.6 | 3.9 ± 0.2 | 14.3 ± 20.5 | 3.8 ± 0.2 |
| **GLOB** | 1.2 to 2.2 | 1 ± 0.4 | 1.2 ± 0.1 | 1.2 ± 0.2 |
| **Na** | 110 to 195 | 153.3 ± 2.6 | 151.3 ± 11.1 | 153 ± 4.3 |
| **K** | 4 to 10.5 | 8.3 ± 0.4 | 7.8 ± 10.8 | 8.5 ± 0 |
| **Cl** | NR | 112 ± 2.2 | 110.5 ± 3.7 | 111.8 ± 3.7 |
| **TCO2** | NR | 26.3 ± 2.4 | 24.5 ± 3.3 | 27 ± 2.6 |

**Table S4. Blood analysis from xenograft study.** NOD-SCID mice were inoculated with MM.1S cells in the flank and treated with PBS (control), RAM2061, bortezomib (Bor), or a combination of RAM2061/Bor. Data are shown as average ± stdev (n=8 per group). Blood was collected at time of euthanasia. Abbreviations: blood urea nitrogen (BUN), creatinine (CRE), alanine amino-transferase (ALT), alkaline phosphatase (ALP), aspartate aminotransferase (AST), total bilirubin (TBIL), glucose (GLU), total protein (TP), albumin (ALB), globulin (GLOB), and NR (not reported).
